# The effect of cash transfers on mental health: Opening the black box – A study from South Africa

**DOI:** 10.1016/j.socscimed.2020.113181

**Published:** 2020-09

**Authors:** Julius Ohrnberger, Laura Anselmi, Eleonora Fichera, Matt Sutton

**Affiliations:** a School of Public Health, MRC Centre for Global Infectious Disease Analysis, Imperial College London, United Kingdom; b Division of Population Health, Health Services Research & Primary Care, University of Manchester, United Kingdom; c Department of Economics, University of Bath, United Kingdom

**Keywords:** South Africa, Poverty, Mental health, Cash transfer, Mediation analysis, Panel data

## Abstract

There is a gap in the literature in understanding how cash transfer programmes affect mental health. We aim to fill this gap by conceptualising and estimating the mediation effects of an unconditional cash transfer programme on mental health. We use a sample of 4,535 adults living below the South African poverty line in four waves (2008–2014) of the South African National Income Dynamics Study. We use information on individual exposure to South Africa's largest unconditional cash transfer programme, the Child Support Grant. Mental health is measured by the 10-item version of the Centre for Epidemiological Depression Scale. We use the product of the coefficient method for the mediation analysis in combination with instrumental variable estimation. We find that physical health and lifestyle factors mediate the relationship of the unconditional cash transfer programme, each explaining about eight percent and 16% of the total positive effect. Our findings show that individuals living in poverty make investment decisions that are positive for their mental health, which has strong implications for policy makers.

## Highlights

• We build a mediation framework for the cash transfer and mental health relationship.
• Analyse mediation effects of the Child Support Grant (CSG) on adult mental health.
• The cash transfer improves mental health by 0.7 units or 0.2 standard deviations.
• Physical health and lifestyle factors mediate the CSG effect on mental health.
• Physical health explains 8% and lifestyle factors explain 16% of the total effect.

## 1 Introduction

The burden of depression in South Africa is about 1.5 times higher than in other Low- and Middle-Income Countries (LMICs) (Jack et al., 2014). Depression is the country's second largest contributor to the burden of disease after HIV and the largest contributor among mental and neural disorders (Jack et al., 2014). Alongside the mental health burden exists a financial burden with about every second person living in poverty. This makes South Africa a particularly important country for the analysis of cash transfer effects on mental health. Few studies have analysed the effects of cash transfers on mental health outcomes and most of these studies look at the direct effects of cash transfers on adolescent, child and maternal mental health and find mixed evidence (Ozer et al., 2011; Baird et al., 2013; Haushofer and Shapiro, 2013; Kilburn et al., 2016, 2019; Angeles et al., 2019; Ohrnberger et al., 2020). Whilst understanding the general direction and magnitude of the effect is important, it is also vital for mental health policies that focus on the poor populations in LMICs to understand the underlying mechanisms (mediators) through which cash transfer programmes affect mental health.

Only three studies in the domain analysed mediation effects of cash transfers on mental health. Ozer et al. (2011) find that 33% of the direct effect of the Mexican conditional cash transfer programme *Opportunidades* on maternal depression was mediated by perceived stress levels. Baird et al. (2013) find that the positive effect of the Zomba Cash transfer programme for Malawian schoolgirls on mental health is mediated by several factors in both their unconditional cash transfer (UCT) and conditional cash transfer (CCT) treatment arms. The CCT effect is fully mediated and the UCT effect is partly mediated by consumption, social interaction and self-rated health. Angeles et al. (2019) estimate the mediation effects of an unconditional cash transfer in Malawi on youth mental health finding full mediation through education, social support and caregiver wellbeing. All three studies have limitations. (i) None of the studies build the analysis on a clearly structured mediation framework, potentially omitting other relevant mediators. (ii) The methodologies applied in the studies do not account for mediator inter-relations, which is essential for the identification of mediation effects. (iii) Findings are limited to the adolescent and maternal sub-population and not applicable to the wider population.

We aim to open the ‘black box’ of the effect of cash transfers on mental health by firstly setting up a mediation framework and secondly using the framework to estimate the mediated effects of the South African Child Support Grant (CSG) on mental health of the poor South African adult population. We use exploratory factor analysis to determine mediator dimensions and use the product of the coefficient method for the mediation analysis following Baron and Kenny (1986). The CSG is a nationwide unconditional cash transfer programme eligible for care takers of children at specific ages that live in economic poverty. We build on previous analysis in Ohrnberger et al. (2020) finding strong positive direct effects of the CSG on adult depression. We use non-experimental data on 4,535 poor individuals from four waves of the South African National Income Dynamics Study (2008–2014). To address self-selection into the programme, we instrument programme receipt with a binary variable measuring if the household has an age-eligible child.

## 2 General mediation framework

We conceptualise in this section a mediation framework of the cash transfer and mental health relationship We build on the Grossman model of health (Grossman, 1972) and its extensions, and on findings from the empirical literature.

### 2.1 The Grossman model of health and extension of the model

The Grossman model of health focuses on individual inter-temporal choices to maximise utility (Grossman, 1972). Ohrnberger et al. (2017) suggest that this model should encompass not just physical, but also mental health. Mental health has a dual role as it has a direct effect on utility but also because healthy time is an input for income generating work (e.g. health capital). Current mental health is explained by a past stock of depreciated mental health and investments in mental health, such as medical care and time spent in health supporting activities (Grossman, 1972; Wagstaff, 1986). Medical care may be a relatively less important health input in the South African context due to resource scarcity, as there is less than one psychologist available per 100,000 uninsured population, although it may vary across the population and localities (Docrat et al., 2019). Health investments could act as mediators of the cash-transfer-mental health relationship. Extensions of the Grossman model include further channels such as social capital and socio-economic status (Bolin et al., 2003; van Kippersluis and Galama, 2013). The original Grossman model and its extensions point to four possible mediation channels of the unconditional cash transfer effect on mental health: social capital, socio-economic status, lifestyle choices, and physical health. Individuals can make investment choices in all four dimensions in order to improve their long term (mental) health.

### 2.2 Empirical findings

#### 2.2.1 Social capital

Social capital has a positive association with good mental health. Lower levels of social capital have been found to predict higher levels of psychological distress (Myer et al., 2008), whereas higher levels of social interaction show a strong positive association with mental health (Dour et al., 2014). Previous research found that cash transfers positively affect social capital (Baird et al., 2013; Owusu-Addo et al., 2018). We identify the following social capital measures and mental health determinants in the literature: social isolation, social class, family contact and family cohesion, or membership of a faith or community group (Jenkins et al., 2008; Lund et al., 2010; Allen et al., 2014). In the context of the Grossman model, individuals may use the cash transfer income to invest into their mental health by trading-off hours worked with time spent socialising. The cash transfer compensates for the opportunity cost of time spent socialising and not working. This effect is also referred to as a negative labour supply effect at the intensive margin and supported by the existing literature on cash transfer (Kabeer and Waddington, 2015; Baird et al., 2018; Porreca and Rosati, 2019).

#### 2.2.2 Socio-economic status

Socio-economic status is a major determinant of mental health (Allen et al., 2014). Higher socio-economic status is found to be positively associated with good mental health (Myer et al., 2008; Lund et al., 2010). Research finds positive effects of cash transfer programmes on socio-economic status (Baird et al., 2013; Owusu-Addo et al., 2018). We identify the following measures for socio-economic status and mental health determinants: household income, food security, employment status, educational attainment, household material resources and financial debt. Income (e.g. aggregate household income and financial resources) and household material resources (assets) show a positive association with good mental health (Patel, 2007; Myer et al., 2008). A negative association with good mental health has been found for unemployment, lower education, financial debt and malnutrition (Patel, 2007; Jenkins et al., 2008; Myer et al., 2008; Lund et al., 2010; Allen et al., 2014). In the Grossman model, cash transfer induced changes in socio-economic status affect the budget constraint. This gives more long-term stability and capabilities for health investments by increased employment or household income and direct investments in health by food security.

#### 2.2.3 Lifestyle choices

Physical activity, dietary choices, smoking, and drinking are lifestyle choices associated with mental health outcomes. Exercising and a healthy diet can significantly reduce the risk of common mental health disorders such as anxiety or depression (Lopresti et al., 2013; Hegberg and Tone, 2015). Substance abuse such as alcohol consumption and smoking can significantly increase the risk of mental health problems and are among the ten leading causes of depression (World Health Organization, 2009). A literature review found strong evidences for good lifestyles among cash transfer recipients (Evans and Popova, 2017). Better lifestyles can be considered within the Grossman model as immediate investments in better present and future health.

#### 2.2.4 Physical health

Physical health is an important determinant of mental health, with better physical health predicting better mental health (Ohrnberger et al., 2017). The literature on cash transfers finds evidence for mostly positive effects of cash transfers on physical health outcomes (Owusu-Addo et al., 2018). In the Grossman model, physical health affects mental health in two ways. Firstly, healthy time is required for income generating activities or time spent on health rewarding activities. Secondly, better health as determinant of mental health has an immediate effect on mental health and thus is a mental health investment.

#### 2.2.5 Living conditions

Although living conditions are not mentioned specifically as determinants of health in the Grossman model or in the extensions, the empirical literature finds that better housing quality, access to water and sanitation and neighbourhood safety are important determinants of good mental health (Gruebner et al., 2012; Roberts and Browne, 2017; Vaid and Evans, 2017). Previous analysis found evidence for positive cash transfer effects on living conditions, such as improved sanitation, housing quality and access to electricity (Akresh et al., 2016; Handa et al., 2016). Living conditions like health investment variables in the Grossman model, are investments in health by the individuals or the household following the increase of the budget constraint due to the cash transfer.

### 2.3 Mediation framework of the mental health and cash transfer relationship

**Fig. 1:**
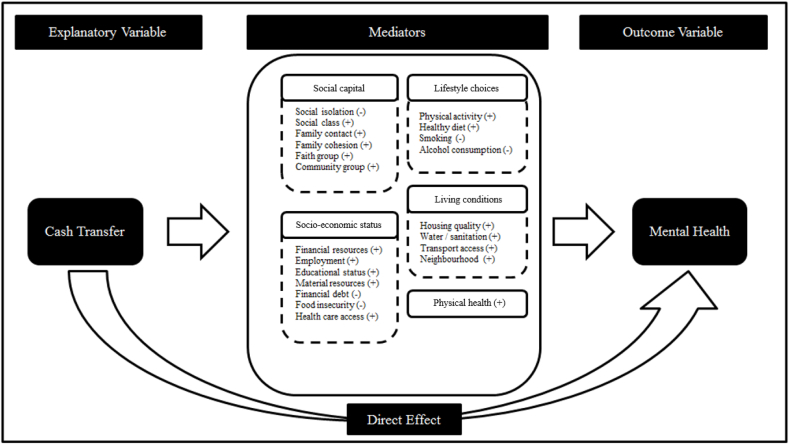
Mediation framework of the cash transfer mental health relationship.

In Fig. 1, we illustrate our hypothesised mediation framework for the cash transfer and mental health relationship. The arrow linking the cash transfer with mental health represents the direct effect. The indirect effects between the two variables are mediators, which are grouped together in the above identified five dimensions. The signs in brackets following each mediator indicate the direction of the hypothesised association of the mediator with mental health.

## 3 Data

### 3.1 The National Income Dynamics Study (NIDS)

We use four waves (2008–2014) of the National Income Dynamics Study which is a representative biennial longitudinal study of the South African population (Southern African Development Research Unit, 2016). We use a sub-sample of lower income groups defined by the 2008 CSG income-eligibility threshold of R800 (US$60) per capita per month. In doing so, we use the sample of poor individuals, as the CSG bound is close to the upper-bound of the national poverty line in 2014 of R779 per month (US$58) (Statistics South Africa, 2014). We exclude individuals that live in CSG receiving households in 2008 and have lived prior to 2008 in a CSG receiving household. We use a baseline of non-CSG recipients in 2008 to avoid reverse causality of the CSG on mediators. We refer to our previous study in which we tested and rejected potential bias due the selection of our study sample from the NIDS sample (Ohrnberger et al., 2020).

### 3.2 The Child Support Grant (CSG)

The South African government established the Child Support Grant (CSG) in 1998. The CSG is the country's largest poverty alleviation programme and represents on average 20–25% of the receiving households' monthly income (Gomersall, 2013). Eligibility to receive the grant is dependent on, firstly, having or taking care for a child in the eligible age-range and, secondly, on means-tested income and assets of the applicant (Delaney et al., 2008). Care takers need to apply for the CSG which leads to a selection problem. Studies have estimated that about 30% of all eligible children are not receiving the grant, due to lack of information, access or motivation (Delaney et al., 2008; Gomersall, 2013).

Initially only designed to include children up to age seven, the South African Government raised the age-eligibility of children for the programme to age 18 over time. The means tested income threshold was also lifted to reflect price-inflation and to make the programme available for the wider poor population of South Africa (Gomersall, 2013). Table A1 in the online appendix illustrates the evolution of the CSG over time.

Other financial support programmes are available and can be jointly received with the CSG upon application. These programmes are the Disability Grant (DG), Old Age Pension (OAP), Foster Care Grant (FCG), and the Child Dependency Grant (CDG) and the War Veteran Pension (WVP) (South African Government, 2004). However, none of these programmes is as large as the CSG.

### 3.3 Outcome, explanatory and control variables

As mental health outcome variable we use the Centre for Epidemiological Studies Depression Scale (CES-D). CES-D is a clinically validated measure for depression and was developed by Radloff (1977). CES-D has been used in a wide set of longitudinal studies and is a validated measure of depression for the poorer populations living in LMICs (Ali et al., 2016). The CES-D is calculated by summing over the scores of 10 items and ranges from zero indicating highest depressive symptoms (worst mental health) to 30 with no depressive symptoms (best mental health).

The main explanatory variable of interest is a binary variable taking value (1) if individuals live in a household in which at least one person receives the CSG grant and value (0) otherwise. This information is based on individual reports of CSG receipt in the household survey. We use a binary instrumental variable for the self-reported CSG receipt at the household level, which takes value (1) if the individual lives in a household which has at least one age eligible child and (0) otherwise.

In the estimations we control for the following variables measured at baseline: a set of binary indicators showing if the individual lives in a household that receives financial support from other governmental programmes (Old Age Pension, Disability Grant, Foster Care Grant or Care Dependency Grant), his/her gender, age, if the individual is younger than 19 years as individuals of up to age 18 fall into the eligible child category of the CSG from 2012 onwards, and a binary variable indicating if the individual is involved in the economic decision making of the household. We also control for the size of the household, a set of regional indicators, if the individual lives in a rural, tribal or urban setting, and a set of province indicators.

### 3.4 Proposed set of mediating variables

We present in this section our applied mediating variables. The variables are grouped by their five hypothesised mediation dimensions.

#### 3.4.1 Measures of social capital

We use as measure for social class the respondent's self-perceived position on the social ladder, ranging from poorest (value 1) to richest (value 6) in the country. We use a binary variable indicating whether a household member has died in the past 2 years as proxy for negative effects on family cohesion, family contact and increased social isolation (Steptoe et al., 2013; Muennig et al., 2018). Social integration is measured by individual preferences to continue living in the current area. The measure ranges from (1) strong preference to stay to (5) strong preference to leave. To proxy the membership to a faith group, we use a variable indicating the importance of religious activities in the respondent's life which takes value (1) if not important at all, (2) if unimportant, (3) if important, and (4) if very important. Other information about individual group memberships is not available in all waves of the NIDS.

#### 3.4.2 Measures of socio-economic status

Educational status is measured by the highest achieved level of education taking on value (0) for no education, value (1) for primary education, value (2) for secondary education, and value (3) for higher education. To measure material resources, we use a 5-item material resource index following Bradley and Putnick (2012). The index is constructed by summing over the following five binary items: ownership of radio, television, telephone, non-human powered transportation, and access to electricity in the household. The index ranges from (0) lowest observed material resources to (5) highest observed resources. Financial resources, food insecurity and employment effects are proxy-measured by the log of the Consumer Price Index adjusted monthly per capita household consumption, which is derived as sum over both food and non-food related expenditures and also includes home-grown products.

We do not include specific measures of investments in health or education and financial debt as these variables have low variation, very low mean values and higher frequencies of non-responses in the NIDS (Daniels et al., 2014).

#### 3.4.3 Measures of lifestyle choices

We measure physical activity with a categorical variable of weekly physical activity, taking value (0) for never physically active, value (1) for less than once a week, value (2) for once or twice a week, and value (3) for three or more times a week. Smoking is measured by the average of daily smoked cigarettes, with value (0) for non-smokers. Drinking is measured by the average number of standard drinks a week, with value (0) for non-drinkers.

A measure for a healthy diet is not included as we do not have information on all individual choices of fruit and vegetable consumption throughout the NIDS survey rounds. Instead we use a binary variable indicating whether the individual is underweight. We do not use the full BMI or different categories of the BMI because of the non-linear effects of BMI levels on health related outcomes (Patra et al., 2016; Zaccardi et al., 2017). The BMI of the respondent is computed as weight in kilograms divided by height in metres squared. Individuals are categorised as underweight with a BMI less than 18.5 kg/m^2^ (World Health Organization, 2000). BMI-levels have been shown to be good proxies for lifestyle choices as they reflect the consumption of unhealthy goods and thus the outcome of dietary choices of an individual (Fuglestad et al., 2012). We compare the category of underweight to all others as previous research found strong associations of being underweight with lifestyle choices such as significantly higher odds to smoke cigarettes (Wang, 2015; Little et al., 2016).

#### 3.4.4 Measures of physical health

We include three measures of self-reported physical health: 1) the number of chronic health conditions (heart problems, asthma, stroke, cancer, high blood pressure and diabetes), with value (0) if the respondent has not recorded a chronic condition; 2) the number of symptoms of illness recorded in the past 30 days with value (0) if the individual did not experience any symptoms and 3) whether the individual is HIV-positive. Symptoms of illness include fever, persistent cough, cough with blood, chest pain, body ache, headache, back ache, joint pain/arthritis, diarrhoea, painful urination, swelling ankles, and severe weight loss.

We include the number of chronic health conditions to measure associations with long-standing health problems. The symptoms of illness are included to account for short-term changes in health. A similar measure has been used in a previous cash transfer analysis (Baird et al., 2013). HIV is included as it is the main burden of disease in South Africa (World Health Organization, 2015) and has been analysed as health outcome in previous cash transfer analysis (Owusu-Addo et al., 2018). All three measures of self-reported physical health show strong negative associations with better mental health (Collins et al., 2006; Steptoe, 2006).

#### 3.4.5 Measures of living conditions

We use two binary variables to measure the quality of housing: 1) a binary variable indicating whether the walls of the house/dwelling are built of cement or bricks, and 2) a binary variable indicating whether the roof of the house/dwelling is covered with cement/bricks/tiles. These measures are also used as proxy for socio-economic status where income or expenditure reliable measures are missing (Akresh et al., 2016; Handa et al., 2016).

The quality of water access is measured by WHO and UNICEF drinking water ladder (World Health Organization and UNICEF, 2008). The drinking water ladder is coded as (1) unimproved, which includes unprotected water sources such as springs, wells, dam/pool/stagnant water, flowing water/stream, and water-carrier/tank; (2) improved, which includes public taps, boreholes, and rain-water tank; and (3) piped, which includes piped water access in the dwelling or on site.

We use the WHO and UNICEF derived sanitation ladder to measure the quality of sanitation in the household (World Health Organization and UNICEF, 2008). The sanitation ladder is coded as follows: (1) open defecation (none); (2) unimproved (bucket toilet and pit latrine without ventilation pipe); (3) shared improved (flush toilet with/without onsite disposal, chemical toilet, pit latrine with ventilation pipe); (4) unshared improved.

Neighbourhood quality is proxy-measured by how common burglaries, muggings, or theft in the neighbourhood are, ranging from (0) never happens to (4) very common.

### 3.5 Descriptive statistics

Table 1, Table 2 show the descriptive statistics for the full pooled sample of individuals living in CSG receiving and non-receiving households. In Table 2, the CES-D takes an average value of 20.1 for the full sample over the three waves indicating moderate mental health. 70% of individuals live in a household with a CSG eligible child whereas only 55% report to live in a CSG receiving household. About every third individual is involved in the economic decision making of a household. About 65% of the sample are female. The average respondent is about 37 years of age and lives with three to five other household members. About a third of individuals live in a household which receives the Old Age Pension and 10% live in a household which receives the Disability grant. Only a small proportion of individuals live in a household receiving Foster Care Grant or Care Dependency Grant.

**Table 1:** Descriptive statistics for the outcome, factors, and baseline covariates by samples.

	Full Sample	CSG Receiving	Non-Receiving CSG
	Mean (SD)	Mean (SD)	Mean (SD)
CES-Depression Scale	20.102 (4.16)	20.397 (4.041)	19.743 (4.275)
HH (Household) receives the CSG	0.549	1	0
HH with eligible child	0.709	0.996	0.36
***Factors identified through factor analysis using waves 2, 3 and 4***			
Living Conditions	2.425 (0.624)	2.389 (0.613)	2.47 (0.633)
Socio economic status	3.942 (0.633)	3.959 (0.59)	3.921 (0.68)
Physical health	0.64 (0.708)	0.654 (0.67)	0.623 (0.751)
Lifestyle	1.236 (2.161)	1.053 (1.887)	1.459 (2.435)
***Control variables at first wave (baseline)***			
Economic decision maker	0.394 (0.489)	0.363 (0.481)	0.431 (0.495)
Male	0.362	0.293	0.445
Age	36.835 (18.139)	35.127 (17.761)	38.915 (18.381)
Age under 19 years	0.175	0.192	0.153
Household members	4.42 (2.224)	4.87 (2.272)	3.872 (2.034)
HH receives Old Age Pension	0.305	0.284	0.331
HH receives Disability Grant	0.119	0.127	0.109
HH receives Foster Care Grant	0.04	0.048	0.03
HH receives Care Dependency Grant	0.011	0.01	0.013
Tribal authority area	0.487 (0.5)	0.518 (0.5)	0.448 (0.497)
Urban formal	0.341 (0.474)	0.308 (0.462)	0.381 (0.486)
Urban informal	0.073 (0.26)	0.071 (0.257)	0.075 (0.264)

Standard deviations in parenthesis. The sample sizes are as follows: Full Sample n = 4,535, CSG receiving n = 2,490, CSG non -receiving n = 2,045.

**Table 2:** Descriptive statistics of proposed mediating items by groups.

	Receiving	Non-Receiving
	Mean (SD)	Mean (SD)
Social ladder	2.354 (0.915)	2.266 (0.977)
Death of HH-member past 2 years	0.145	0.115
Importance of religious activities	3.385 (0.683)	3.317 (0.754)
Area preferences	1.666 (1.077)	1.741 (1.147)
Education	1.461 (0.745)	1.399 (0.74)
Material index	3.068 (1.184)	2.927 (1.249)
Monthly per capita HH-consumption (log)	7.148 (0.678)	7.186 (0.77)
Physical activity	0.444 (0.935)	0.595 (1.063)
Number of cigarettes smoked daily	1.331 (4.073)	1.897 (4.617)
Number of units of alcohol drunk daily	1.341 (5.29)	2.095 (7.412)
Underweight (Body Mass Index < x)	0.049	0.083
Number chronic health conditions	0.261 (0.563)	0.339 (0.667)
Symptoms of illness past 30 days	1.375 (1.839)	1.501 (1.988)
HIV	0.023	0.021
Walls cement/bricks	0.494	0.531
Roof cement/bricks/tiles	0.059	0.06
Water ladder	2.455 (0.723)	2.561 (0.671)
Sanitation ladder	3.037 (1.03)	3.126 (0.973)
Frequency of crimes in neighbourhood	2.061 (1.426)	2.047 (1.426)

Standard deviations in parenthesis. The sample sizes are as follows.

CSG receiving n = 2,490, CSG non -receiving n = 2,045.

Table 2 presents the descriptive statistics of the 19 proposed mediating items. The lifestyle and physical health items are higher for recipients. Average weekly physical activity is higher in the group of CSG receiving households. Non-recipients have, on average, twice as high number of daily smoked cigarettes, higher average weekly alcohol consumption and relatively higher percentage of underweight. The average numbers of chronic conditions and illness symptoms recorded in the past 30 days is higher for non-recipients.

## 4 Empirical framework

### 4.1 Direct treatment effect estimation

To address self-selection in the CSG programme, we use 2SLS estimation. We instrument the binary treatment variable with the binary variable indicating if the individual lives in a household which is eligible to receive the grant. The instrumental variable estimation of the direct effect follows Ohrnberger et al. (2020). We refer to their study for a detailed discussion on the plausibility of the instrumental variable assumptions.

The first stage estimation is formalized in equation (1)

(1) $$CSG_{i,t}=c_{0}+c_{1}Z_{i,t}+X_{i,t=1}c_{2}+t_{t}+R_{t=1}+v_{i,t}$$

where $CSG_{i,t}$ is the binary variable indicating if an individual *i* lives in a household that receives the child support grant in waves two (2010), three (2012) and four (2014). $Z_{i,t}$ is the instrumental variable. $X_{i,t=1}$ are individual characteristics at wave one before individuals in our sample received the CSG. $t_{t}$ are year effects $R_{t=1}$ are regional fixed effects associated with the region of residence at baseline (wave one in 2008) the baseline and $v_{i,t}$ is the individual error term.

In the second stage, we regress mental health (CES-D) on the linear prediction of $CSG_{t}$ from the first stage:

(2) $$CES_{i,t}=\beta_{0}+\beta_{1}\hat{CSG_{i,t}}+X_{i,t=1}\beta_{2}+t_{t}+R_{t=1}+\varepsilon_{i,t}$$

Using a binary variable as outcome variable in the first stage with a binary variable as an instrument, we estimate the Wald-estimator conditional on covariates (Angrist and Pischke, 2008).

### 4.2 Mediation analysis

For the mediation analysis we use the product of the coefficient method by Baron and Kenny (1986). The product of the coefficient is the coefficient of treatment on the mediator multiplied by the coefficient of the mediator on the outcome and is a common approach used in mediation analysis (Keele et al., 2015). As proposed by Yamamoto (2014), Frölich and Huber (2015), and Keele (2015), confounding in treatment with mediation analysis can be address by using instrumental variable estimation. In doing so, we require the exclusion assumption also to hold for the relationship between the instrumental variable and the mediator. We assess this assumption in the robustness test section.

We estimate two equations to identify a mediator in the effect of the treatment on mental health.

Firstly, treatment (receiving CSG) must statistically significantly affect the mediator,

(3) $$M_{i,t}=\gamma_{0}+\gamma_{1}\hat{CSG_{i,t}}+X_{i,t=1}\gamma_{2}+t_{t}+R_{t=1}+\lambda_{i,t}$$

Equation (3) is the second stage of the instrumental variable estimation, where $M_{i,t}$ is the mediator and where controls for baseline factors $X_{i,t=1}$ and year effects are included. The first stage remains the same equation as for the direct effect estimation.

Secondly, the mediator has to statistically affect the outcome (CES-D) which is expressed in equation (4)

(4) $$CES_{i,t}=\alpha_{0}+M_{i,t}\alpha_{1}+X_{i,t=1}\alpha_{2}+t_{t}+R_{t=1}+\rho_{i,t}$$

where $M_{i,t}$ is a vector of mediators. M can be considered a mediator of the relationship only if there are significant effects of $\gamma_{1}$ in (3) and significant effects of $\alpha_{1}$ in (4) (Keele, 2015). Equation (4) is estimated for each mediator separately. The product of the coefficient is then given by ($\alpha_{1}\text{*}\gamma_{1}$), which is the effect of treatment on the mediator times the effect of the mediator on the outcome. We use bootstrapping with 500 repetitions to compute standard errors and confidence intervals.

The identification of the effect through a mediator requires the sequential ignorability assumption which is: 1) No confounding of the treatment-mediator relationship and of the treatment-outcome relationship; 2) No confounding of the outcome-mediator relationship by treatment or other characteristics; 3) No post-treatment confounding of mediators (Imai et al., 2011). In addition, the conditional expectations of the mediators and the outcome have to be both linear (continuous) and additive and the mediators and treatment have to be measured without error (Keele et al., 2015). Another concern of multiple-mediator estimation is that collinearity may arise the more mediators are included, causing a lack of precision due to inflated standard errors of the estimation.

### 4.3 Exploratory factor analysis (EFA) to define mediator dimensions

We use exploratory factor analysis (EFA) to identify the five hypothesised mediating dimensions of our mediation framework, and to solve issues of multiple-mediator estimation. By using EFA, we increase the plausibility of the sequential ignorability assumption as: 1) EFA reduces the data and by doing so it reduces the issue of multiple correlations and moderation effects between the mediators, thus makes the inter-relations of the mediators testable; 2) EFA generates continues scale variables and solves the issue of the different non-normal scales of mediators; 3) EFA improves the precision as it reduces the elements included in the estimation and reduces omitted variables bias and measurement error in the mediators (Heckman et al., 2013). We compute the factors on three waves of data, 2010–2014 excluding the baseline in 2008 as we do not use mediators at baseline for the main analysis. A technical discussion of EFA is provided in the online appendix.

## 5 Robustness analysis

### 5.1 Assessing the sequential ignorability assumption

We use four tests to assess the plausibility of the sequential ignorability assumption. Firstly, we control for baseline levels of the observed mediators. Secondly, we test if errors of the outcome equation are correlated with errors of the mediator equations (Keele et al., 2015). These two tests should give insight about bias in the estimation due to confounding factors and violation of part 2) of the sequential ignorability assumption. A third test relates to the assumption of no post-treatment confounding in the mediators. We test if interaction effects of the identified mediators on the outcome are present. The fourth test relates to the non-confounding assumption in the mediator-outcome relationship. We regress CES-D on treatment interacted with identified mediators.

### 5.2 Addressing the exclusion restriction of the instrumental variable

The instrumental variable estimation within mediation analysis requires that mediators are only affected by the instrumental variable through the instrumented variable. We use a placebo-test to identify if having an eligible child or living in a household with an eligible child has different implications on the set of identified mediators than having no eligible child or living in a household without an eligible child. We use observations of individuals at baseline and regress the mediator on a dummy variable indicating if the individual lives in a household that will receive the CSG in the following wave. No significance of the dummy variable would imply that differences in having a child or having a child close to the eligibility age would not affect the mediators.

We also re-estimate the direct effect equation (2), mediator equation (3), mediator-outcome equation (4) including dummy variables that indicate the age of the children living with the individual in the household. If the results remain similar to the results without the dummy variables controlling for child-age effects, this suggest that the instrumental variable indeed only affects mental health and the mediators through the instrumented variable.

### 5.3 Test of attrition effects

Individuals are observed at least twice and at most four times in our sample. To test possible attrition effects, we code three binary variables. A first binary outcome variable takes value one for individuals that are observed at any point and zero otherwise. A second binary outcome variable takes value one for individuals that are observed at baseline and twice thereafter and zero otherwise. A third binary outcome variable takes value one for individuals that are observed at baseline and once thereafter and zero otherwise. We estimate two logistic regression models to identify if differences in observations are conditional on: i) baseline characteristics; or ii) levels of the mediating variables at baseline.

## 6 Results

### 6.1 Direct treatment effect estimation and attrition tests

Table 3 presents the results from the first and second stage of the instrumental variable estimation of the effect of living in a CSG receiving household on adult mental health. The first stage results show that the instrumental variable has strong relevance, given the high magnitude of the estimated effect (0.747) on receiving a CSG and the statistical significance.

**Table 3:** First and second stage regression of the direct effect of the CSG on mental health.

	(1)	(2)
	1st Stage: Direct effect	2nd Stage: Direct effect
HH receives CSG		0.823*** (0.223)
HH with CSG eligible child	0.747*** (0.014)	
Economic decision maker	0.006 (0.014)	−0.273* (0.153)
Male	−0.024** (0.011)	0.324** (0.126)
Age	−0.006*** (0.002)	−0.079*** (0.021)
Age Squared	0.000** (0.000)	0.001*** (0.000)
Under 19 years of age	−0.027 (0.021)	0.444** (0.193)
HH receives Old Age Pension	−0.017 (0.019)	−0.003 (0.181)
HH receives Disability Grant	0.034 (0.024)	−0.386* (0.234)
HH receives Foster Care Grant	0.010 (0.041)	0.315 (0.323)
HH receives Care Dependency Grant	−0.171*** (0.066)	0.361 (0.662)
Number of HH members	−0.005 (0.005)	0.014 (0.037)
Constant	0.097* (0.056)	22.506*** (0.626)
Province and region at baseline	YES	YES
Year	YES	YES
Observations	4,535	4,535
R-squared	0.521	0.065

Clustered standard errors in parentheses; ***p < 0.01, **p < 0.05, *p < 0. The outcome variable in the first stage is the binary variable HH CSG. The outcome variable in the second stage is CES-D.

The second stage estimation shows that CSG-receipt has a positive strong and significant effect of 0.823 or about one quarter of a standard deviation in mental health. Males show better mental health compared to females. Age has a negative non-linear association with mental health with the turning point at age 40 and positive associations for individuals below age 19. Being the economic decision maker and living in a household with a Disability Grant recipient has negative effects on mental health.

### 6.2 Exploratory factor analysis to identify conceptualised mediating dimensions

Table A4 in the online appendix presents the main findings from the exploratory factor analysis. We refer to the online appendix for a complete stepwise discussion of the exploratory factor analysis.

Following the literature, we use items with factor loadings greater than 0.32 to identify the dimension of each retained factor (Tabachnick and Fidell, 2007). The first factor which has the highest eigenvalue loads on material index, walls and roof quality, water and sanitation ladder. We interpret this factor as the living conditions dimension in the mediation framework outlined in Fig. 1. The second factor loads on social ladder, material index, monthly logged household per capita consumption and roof quality. We interpret this factor as the socio-economic status. The third factor loads on education, physical activity, the number of chronic health conditions and the number of symptoms of illness in the past 30 days and It reflects the physical health dimension. The last retained factor correlates with the number of daily smoked cigarettes and the number of daily units of standard drinks and is a latent variable for lifestyles. All the items defining the four factors have the sign hypothesised by the framework. We do not identify a social capital dimension in the data.

### 6.3 Identification of mediators

We present in Table 4 the four mediator equations, using the four derived factor dimensions, and the outcome equation, to identify significant mediators of the cash transfer mental health relationship.

**Table 4:** Test of mediator identification: Second stage estimations of the instrumental variable estimation of mediator and outcome equations.

	(1)	(2)	(3)	(4)	(5)
	2nd Stage: Living conditions (mediator)	2nd Stage: Soc.-econ. Status (mediator)	2nd Stage: Physical Health (mediator)	2nd Stage: Lifestyles (mediator)	2nd Stage: CES-D (outcome)
HH receives CSG	−0.025 (0.035)	0.091** (0.042)	0.062* (0.032)	−0.415*** (0.113)	0.710*** (0.213)
Living conditions					0.647*** (0.184)
Soc.-econ. status					−0.101 (0.133)
Physical health					1.020*** (0.139)
Lifestyles					−0.182*** (0.042)
Economic decision maker	−0.051** (0.023)	0.011 (0.026)	0.014 (0.024)	−0.117 (0.096)	−0.275* (0.150)
Male	0.166*** (0.018)	−0.137*** (0.023)	0.269*** (0.019)	1.233*** (0.087)	0.154 (0.129)
Age	0.005 (0.004)	−0.000 (0.004)	−0.036*** (0.004)	0.038*** (0.011)	−0.039* (0.021)
Age Squared	−0.000 (0.000)	0.000 (0.000)	0.000*** (0.000)	−0.000*** (0.000)	0.001** (0.000)
Under 19 years of age	−0.005 (0.035)	0.059 (0.038)	−0.135*** (0.026)	−0.321*** (0.114)	0.533*** (0.189)
HH receives Old Age Pension	−0.015 (0.029)	0.004 (0.032)	0.017 (0.026)	−0.095 (0.087)	−0.028 (0.180)
HH receives Disability Grant	−0.019 (0.039)	0.042 (0.044)	−0.144*** (0.039)	−0.104 (0.095)	−0.242 (0.230)
HH receives Foster Care Grant	0.066 (0.078)	0.083 (0.057)	0.063 (0.041)	0.017 (0.150)	0.219 (0.327)
HH receives Care Dependency Grant	−0.078 (0.091)	0.073 (0.141)	−0.053 (0.078)	−0.759*** (0.189)	0.335 (0.645)
Number of HH members	0.017** (0.007)	0.030*** (0.007)	0.012** (0.005)	−0.044*** (0.016)	−0.014 (0.036)
Constant	2.424*** (0.114)	3.566*** (0.114)	1.710*** (0.089)	0.550* (0.282)	19.656*** (0.862)
Province and region at baseline	YES	YES	YES	YES	YES
Year	YES	YES	YES	YES	YES
Observations	4,535	4,535	4,535	4,535	4,535
R-squared	0.355	0.129	0.436	0.152	0.095

Clustered standard errors in parentheses; ***p < 0.01, **p < 0.05, *p < 0. We present the second stage estimation of the instrumental variable estimation. The instrumented variable is HH receives CSG which is instrumented by HH with an eligible child.

Results from the second stage estimations with the mediators as outcome variables in columns (1) to (4) presented in Table 4 show that socio-economic status, physical health and lifestyles are significantly associated with the instrumented household receipt of the CSG. CSG receipt improves socio economic status, physical health and induces better lifestyle choices. Living conditions are not significantly affected by CSG receipt.

The second stage regression in column (5) shows a strong and significant effect of CSG receipt. However, the effect is reduced compared to the direct effect presented in Table 3 due to mediation effects. The following factors are statistically significantly associated with CES-D: living conditions with positive effects (0.65) of a fifth of a standard deviation in CES-D; physical health with a strong positive effect (1.02) of about a quarter of a standard deviation in CES-D; and lifestyles with a negative effect (−0.182). Socio economic status does not show a significant association. Lifestyles and physical health are identified as two mediators as they are significantly affected by the treatment and significantly affect the outcome.

### 6.4 Product of the coefficient approach

Table 5 presents the findings from the mediation analysis. Significant positive mediation effects of the cash transfer mental health relationship are identified for both mediators. Physical health explains 0.063 units of the cash transfer effect on mental health and lifestyle factors explain 0.076 units of the cash transfer effect on mental health. This implies that an individual that lives in a cash transfer receiving household has improved physical health due to the transfer, and these improvements then improve mental health.

**Table 5:** Mediation effect of lifestyle factors and physical health in the CSG - CES-D relationship.

	Effect of CSG on CES-D	Percent of Total Effect
A: Mediated physical health	0.063* (0.035)	7.4
B: Mediated lifestyles	0.076*** (0.026)	8.9
C: Indirect effect (A+B)	0.139*** (0.045)	16.3
D: Direct effect	0.71*** (0.217)	83.7
E: Total effect (C+D)	0.849*** (0.222)	100

Bootstrapped clustered standard errors in parenthesis***p < 0.01, **p < 0.05, *p < 0.500 bootstrap repetitions. The estimation coefficients are the effect of mediating factors of CSG on CES-D.

For the interpretation of mediated lifestyle effects, we need to consider the negative effects both of treatment on the lifestyles and of lifestyles on the outcome. The findings here imply that an individual residing in a household receiving cash transfer has improved lifestyle factors. These improvements translate into an improvement in mental health of 0.076 units.

Physical health explains 7.4% and lifestyle factors explain 8.9% of the total effect of the cash transfer on mental health. The accumulated indirect effect of the two mediators amounts to 0.139 which is about 16.3% of the total effect. The remaining 83.7% of the total effect is explained by the direct effect.

### 6.5 Robustness tests

Our four tests to assess the sequential ignorability assumption are presented in tables A6 to A9 in the online appendix. All tests provide strong evidence for the sequential ignorability assumption to hold. Including baseline mediator measures, lifestyle choices remain strong mediators (tables A6 and A7). We observe no error-correlation between the mediator and outcome equations which supports the sequential ignorability assumption (table A8). Neither do we observe mediator-moderation effects between the mediators and between the mediators with treatment (table A9). We further find strong evidence to support the exclusion assumption to hold between the mediators and treatment (tables A10 and A11). Our findings from the attrition analysis indicate that our results are robust to attrition effects (tables A12 and A13). We refer for an elaborated discussion of the robustness tests to the online appendix.

## 7 Discussion and conclusion

This is the first study to analyse the mediation effects of a cash transfer on the mental health of the poor adult population in a LMIC. In the analysis we use a sample of 4,535 adults living in financial poverty from four waves of the South African National Income Dynamics Study (NIDS). The NIDS records information on individual and household exposure to the South African Child Support Grant (CSG), an unconditional cash transfer programme, which is used for the analysis. We measure mental health with the Centre for Epidemiological Depression scale (CES-D).

We open the black box of cash transfer effects on mental health by: 1) setting up a mediation framework of the cash transfers and mental health relationship; 2) identifying potential mediator dimensions of the framework using exploratory factor analysis; 3) identifying mediator effects of the CSG on mental health using the product of the coefficient method combined with instrumental variable estimation.

We find a positive and statistically significant direct effect of the cash transfer on mental health equal to 0.71 or equivalent to a fifth of a standard deviation in CES-D. Physical health and lifestyles are identified as significant mediators of the cash transfer and mental health relationship. The mediated effect through physical health has a magnitude of 0.063 or 7.4% of the total effect. Lifestyles mediate an effect size of 0.076 or 8.9% of the total effect. Social capital is not identified in the exploratory factor analysis and living conditions and socio-economic status are not found to be mediators.

Using robustness analysis, we find overall strong support for the sequential ignorability assumption of the mediator system, the exclusion assumption of the instrumental variable with the mediators, and no attrition effects on the estimation.

A limitation of the research is that no social capital dimension is identified in the data and therefore remains untested as a mediator. Previous research showed that social capital variables can mediate the cash transfer and mental health relationship (Baird et al., 2013; Angeles et al., 2019). Another limitation is that we do not have measures of stress or stigma in the data which can be considered pathways of the effect (Baird et al., 2013; Angeles et al., 2019). However, following the social causation hypothesis of mental health disorders, in which poverty increases financial stress, stigma and the risk for depression, such effects should be picked up by the socio-economic pathway (Lund et al., 2018).

We used several hypothesis tests to assess whether the sequential ignorability assumption of our mediators holds. All these test results supported the required assumptions for the validity of the identified mediators. However, a limitation of these tests is the small sample size of this study which can statistically under-power the hypothesis tests. Future research should apply the mediation framework to other common mental health disorders, as poverty can affect different mental health disorders in different ways (Burns, 2015), and explore how the mediation framework applies at different severity levels.

Our study showed that unconditional cash transfers have the potential to improve mental health in low-income populations through life-style and physical health pathways. Expanding or offering cash transfers to currently unreached populations in LMICs could thus improve mental health through similar pathways. The analysis identified only positive mediator effects on mental health and no unwanted negative side-effects of the unconditional cash transfer on mental health, requiring neither behavioural change nor giving any information to the recipients on behaviours. Poor individuals invest the marginal income gain in better lifestyles and better physical health which translates into better mental health. The improvements in the lifestyles and physical health, such as reduced symptoms of illness or more physical activity, then translate into improvements in mental health, which affects working time and income generating activities positively. Thus, this analysis of unconditional cash transfer also helps in understanding how individuals invest in mental health. This can have important implications for both mental health and anti-poverty policies in LMICs.

## Author contribution

Julius Ohrnberger: Conceptualization, Methodology, Software, Validation, Formal analysis, Data curation, Writing - original draft, Visualization Eleonora Fichera: Conceptualization, Methodology, Validation, Writing - review & editing Matt Sutton: Conceptualization, Methodology, Validation, Writing - review & editing Laura Anselmi: Conceptualization, Methodology. Validation, Writing - review & editing.
