# Supplementary material for: The effect of cash transfers on mental health: Opening the black box – A study from South Africa
Source: Soc Sci Med. 2020 Sep;260:113181. doi: 10.1016/j.socscimed.2020.113181 (PMC7441310; doi:10.1016/j.socscimed.2020.113181)
Supplement: Multimedia component 1 [file mmc1.docx]

APPENDIX: *Opening the black box of poverty alleviation programme effects on mental health – A case study from South Africa*

We present in the appendix (1) the different analytical steps of the Exploratory Factor analysis of the four identified mediator dimensions “Socio-economic status”, “Life-style choices”, “Living conditions” and “Physical health”, and (2) findings from the robustness analysis.

# Exploratory factor analysis (EFA)

## Pre-factor analysis tests

Before we start using factor analysis, we have to test if correlations between the variables are strong enough. The Bartlett test of sphericity and the Kaiser-Meyer-Olkin measure of sampling adequacy (KMO) are used to determine whether the correlation of the variables warrants factor analysis.

The Bartlett test of sphericity tests the null hypothesis that the *m* variables are correlated (e.g. it tests the hypothesis that the correlation matrix is an identity matrix) (Bartlett, 1937). The test rejects the null hypothesis with a p-value <0.001 and strongly supports the applicability of factor analysis on the data. The KMO statistics measures how small the partial correlations between each pair of variables are in comparison to the zero-order correlations and takes values between 0 and 1 (Kaiser, 1974; Cerny and Kaiser, 1977). Smaller values indicate that the *m* variables have too little in common to conduct a factor analysis, e.g. that partial correlations are large. The cut-off point is 0.5 (approximate value of the identity matrix), with values >0.5 indicating that the variables have enough in common for a meaningful application of factor analysis (Kaiser, 1974). In our application, the KMO statistics for the variables is 0.677 >0.5. This supports the sample adequacy for factor analysis.

## Findings from the Exploratory factor analysis

Tables A2 to A5 present the results from the exploratory factor analysis. Tables A2 and A3 show the eigenvalues and factor loadings from the unrotated factor analysis. Figure A1 shows the scree-plot of the eigenvalues of the unrotated factor solution, which we use to determine the number of factors to retain. The point of inflexion is visible where the two dashed lines intersect. As suggested in the literature (Tabachnick and Fidell, 2007), we retain the four factors with eigenvalues greater than the inflexion point, implying that these factors add explanatory variation to the full data. Once we retain the four factors we *quartimin* rotate the factor loadings to make sensible interpretations of the dimensions possible.

Table A4 presents the rotated factor loadings. Following the literature, We use items with loadings greater than 0.32 to identify the dimension of each retained factor (Tabachnick and Fidell, 2007; Yong and Pearce, 2013). The first factor which has the highest eigenvalue loads on material index, walls and roof quality, water and sanitation ladder. We interpret this factor as the living conditions dimension in the mediation framework outlined in figure 1 in the paper. The second factor loads on social ladder, material index, monthly logged household per capita consumption and roof quality. We interpret this factor as the socio-economic status. The third factor loads on education, physical activity, the number of chronic health conditions and the number of symptoms of illness in the past 30 days. The third factor hence reflects the physical health dimension. The last retained factor correlates with the number of daily smoked cigarettes and the number of daily units of standard drinks and is a latent variable for life styles.

The communality of the variables, which is calculated as communality = 1 - uniqueness of variance, is satisfactory. The most variation explained by the four factors is for the variable water ladder which has a common variance of 0.56. The least explained variation of a variable by the retained factors is the variable indicating the number of daily units of alcohol with a common variance of 0.13. All of the items defining the four factors have the correct sign as hypothesised by the framework, for example positive associations of physical activity and negative associations of the number of chronic health conditions with the identified physical health factor dimension.

In order to compute the four factor dimensions, we obtain corresponding weights or factor scoring coefficients for the 19 items. Table A5 presents the factor scoring coefficients obtained by regressing the four dimensions on the 19 items. The factor scores are computed by multiplying the corresponding item by the weight and summing over each of the 19 items for each factor. Following the weighting of the items, higher values in physical health, socio economic status and living conditions imply better health, higher socio-economic status and improved living conditions. Higher values in life styles imply worse life style choices due to the positive correlations of the number of alcoholic drinks and the number of smoked cigarettes with the factor.

# *Robustness tests*

Tables A6 and A7 present the first and second stage of the reduced sample estimation including baseline levels of the two mediators. The instrumental variable remains a strong predictor of household eligible for the CSG (0.738 to 0.740). In table A6, the effect of instrumented treatment remains significant for lifestyles with size -0.26 in model (2). While in the outcome equation (6) both mediators keep their significant effects on mental health, with physical health having an effect of size 0.993 or a five per cent change from the mean and life styles having an effect of size -0.161 or a one per cent change from the mean.

Table A8 presents the test of error-correlations of residuals from the mediator equations with residuals from the outcome estimation. No error-correlation is observed which supports the sequential ignorability assumption.

Table A9 presents our findings from the mediator-moderator analysis. We find no joint significant interaction effect on CES-D, neither between the identified mediators life-styles and physical health nor between the identified mediators and treatment status. These findings support the sequential ignorability of the mediators and the underlying assumption of similar effects of mediators on the outcome.

Two tests on the exclusion assumption of the instrumental variable with the mediators are presented in tables A10 and A11. The second stage estimates of the placebo-dummy estimation are presented in table A10. Results show no significant effect of the instrumented household receipt of CSG receipt in subsequent waves on the mediators in (2) and (4). We interpret these findings as support for no anticipation effects but also as support for the causality of the link between treatment and the mediators and the exclusion assumption of the instrumental variable with the mediators.

We present in table A11 the estimations of the direct effect equation, of the mediator equation and of the mediation outcome equation controlling for potential child age effects on mental health. Neither the effects of the mediators on mental health nor the instrumental variable estimation of treatment effects on the mediator and on mental health, are affected in significance or magnitude when controlling for child age effects. This evidence gives further support for the exclusion assumption of the instrumental variable.

We show findings from the attrition analysis in tables A12 and A13. The logistic regression analysis of possible attrition effects by baseline characteristics is presented in table A12. Findings show that the majority of the variables do not predict attrition and we thus conclude that our tests of attrition by different categories and using baseline characteristics indicate that overall the sample remains strong and representative. Table A13 presents the attrition analysis but now conditioning on the suggested mediating variables at baseline. Overall the findings suggest that attrition is hardly affected by baseline levels of the 19 mediator items indicating and that the sample is suited for the analysis.

**APPENDIX: Tables and figures**

Table A1 Child Support Grant age and income eligibility criteria and grant value between 1998 and 2014

| **Legislation date** | **Eligible age (years)** | **Income threshold (South African Rand per month)** | **Grant amount (South African Rand per month)** |
| --- | --- | --- | --- |
| 01/10/1998 | 0-7 | R 800 in Rural Areas;  R 1,100 in Urban Areas  Unchanged until October 2008 | R 100 |
| 01/07/1999 | 0-7 |  | R 100 |
| 01/07/2000 | 0-7 |  | R 100 |
| 01/07/2001 | 0-7 |  | R 110 |
| 01/04/2002 | 0-7 |  | R 140 |
| 01/10/2002 | 0-7 |  | R 160 |
| 01/04/2003 | 0-9 |  | R 160 |
| 01/04/2004 | 0-11 |  | R 170 |
| 01/04/2005 | 0-14 |  | R 180 |
| 01/04/2006 | 0-14 |  | R 190 |
| 01/04/2007 | 0-14 |  | R 200 |
| 01/04/2008 | 0-14 |  | R 210 |
| 01/10/2008 | 0-14 | R 2,300 | R 230 |
| 01/01/2009 | 0-15 | R 2,400 | R 240 |
| 01/04/2010 | 0-16 | R 2,500 | R 250 |
| 01/04/2011 | 0-17 | R 2,600 | R 260 |
| 01/01/2012 | 0-18 | R 2,800 | R 280 |
| 01/04/2013 | 0-18 | R 2,900 | R 290 |
| 01/04/2014 | 0-18 | R 3,100 | R 310 |
| 01/10/2014 | 0-18 | R 3,200 | R 320 |

Source: (Eyal and Burns, 2015): Notes: A.) Age refers to the upper age limit. B.) The income threshold for CSG eligibility was defined as 10 times the grant amount in October 2008 to adjust for constantly increasing price-inflation. C.) If the primary caregiver is married, the income threshold is doubled, for instance to R 6,400 per month in October 2014.

**Table A2 Eigenvalues after principal factor analysis**

|  | **Eigenvalue** | **Difference** | **Proportion** | **Cumulative** |
| --- | --- | --- | --- | --- |
| **Factor1** | 2.177 | 1.01 | 0.582 | 0.582 |
| **Factor2** | 1.167 | 0.492 | 0.312 | 0.894 |
| **Factor3** | 0.675 | 0.233 | 0.180 | 1.075 |
| **Factor4** | 0.442 | 0.163 | 0.118 | 1.193 |
| **Factor5** | 0.279 | 0.089 | 0.075 | 1.267 |
| **Factor6** | 0.19 | 0.013 | 0.051 | 1.318 |
| **Factor7** | 0.177 | 0.105 | 0.047 | 1.365 |
| **Factor8** | 0.072 | 0.028 | 0.019 | 1.385 |
| **Factor9** | 0.044 | 0.017 | 0.012 | 1.396 |
| **Factor10** | 0.027 | 0.045 | 0.007 | 1.404 |
| **Factor11** | -0.018 | 0.037 | -0.005 | 1.399 |
| **Factor12** | -0.055 | 0.018 | -0.015 | 1.384 |
| **Factor13** | -0.073 | 0.059 | -0.020 | 1.365 |
| **Factor14** | -0.132 | 0.029 | -0.035 | 1.329 |
| **Factor15** | -0.161 | 0.033 | -0.043 | 1.286 |
| **Factor16** | -0.194 | 0.062 | -0.052 | 1.234 |
| **Factor17** | -0.256 | 0.03 | -0.068 | 1.166 |
| **Factor18** | -0.286 | 0.049 | -0.076 | 1.089 |
| **Factor19** | -0.335 |  | -0.090 | 1.000 |
| Unrotated principal factors from 4,535 observations with 85 parameters. LR test: independent vs. saturated: chi2(171) = 0.000 Prob>chi2 =0.000 | | | | |

**Table A3 Factor loadings after principal factor analysis**

|  | **Factor1** | **Factor2** | **Factor3** | **Factor4** | **Factor5** | **Uniqueness** |
| --- | --- | --- | --- | --- | --- | --- |
| **Social ladder** | 0.272 | 0.011 | -0.299 | 0.046 | -0.082 | 0.828 |
| **Death of HH-member past 2 years** | -0.021 | -0.020 | -0.088 | 0.045 | -0.079 | 0.983 |
| **Importance of religious activities** | 0.105 | 0.159 | -0.079 | 0.026 | 0.250 | 0.895 |
| **Area preferences** | -0.018 | -0.243 | -0.064 | 0.106 | 0.064 | 0.921 |
| **Education** | 0.405 | -0.502 | -0.147 | 0.123 | 0.066 | 0.544 |
| **Material index** | 0.395 | 0.065 | -0.125 | 0.038 | 0.017 | 0.640 |
| **Monthly per capita HH-consumption (log)** | 0.395 | 0.106 | -0.204 | 0.275 | -0.064 | 0.711 |
| **Physical activity** | 0.231 | -0.333 | 0.035 | 0.216 | 0.182 | 0.755 |
| **Number daily cigarettes** | 0.060 | -0.017 | 0.350 | 0.240 | -0.165 | 0.789 |
| **Number daily units of alcohol** | 0.007 | -0.085 | 0.312 | 0.163 | -0.190 | 0.832 |
| **BMI underweight** | 0.028 | -0.121 | 0.313 | 0.073 | 0.173 | 0.851 |
| **Number chronic health conditions** | -0.043 | 0.611 | 0.006 | 0.047 | 0.048 | 0.620 |
| **Symptoms of illness past 30 days** | -0.093 | 0.444 | 0.072 | 0.206 | 0.166 | 0.719 |
| **HIV** | -0.150 | 0.145 | 0.053 | 0.280 | 0.040 | 0.874 |
| **Walls cement/bricks** | 0.395 | 0.048 | 0.207 | -0.217 | 0.113 | 0.570 |
| **Roof cement/bricks/tiles** | 0.395 | 0.266 | -0.172 | 0.032 | -0.104 | 0.601 |
| **Water ladder** | 0.395 | 0.064 | 0.256 | -0.085 | 0.013 | 0.443 |
| **Sanitation ladder** | 0.395 | 0.107 | 0.148 | -0.078 | -0.087 | 0.706 |
| **Frequency of crimes in neighbourhood** | 0.060 | 0.047 | -0.046 | 0.116 | -0.035 | 0.977 |
| Factor loadings >0.32 are highlighted in grey | | | | | | |

**Table A4 Quartimin rotated factor scores with four retained factors**

|  | **Factor 1 (Living conditions)** | **Facto 2 (Soc. econ. status)** | **Factor 3 (Physical Health)** | **Factor 4 (Life styles)** | **Uniqueness** |
| --- | --- | --- | --- | --- | --- |
| **Social ladder** | -0.012 | 0.339 | 0.108 | -0.178 | 0.835 |
| **Death of HH-member past 2 years** | -0.098 | 0.080 | 0.037 | -0.024 | 0.989 |
| **Importance of religious activities** | 0.039 | 0.151 | -0.114 | -0.048 | 0.957 |
| **Area preferences** | -0.151 | 0.056 | 0.255 | 0.052 | 0.925 |
| **Education** | 0.053 | 0.218 | 0.611 | 0.013 | 0.548 |
| **Material index** | 0.336 | 0.347 | 0.086 | -0.081 | 0.640 |
| **Monthly per capita HH-consumption (log)** | 0.027 | 0.519 | 0.048 | 0.044 | 0.715 |
| **Physical activity** | 0.017 | 0.143 | 0.386 | 0.198 | 0.788 |
| **Number daily cigarettes** | 0.127 | -0.035 | -0.014 | 0.412 | 0.816 |
| **Number daily units of alcohol** | 0.096 | -0.105 | 0.040 | 0.336 | 0.869 |
| **BMI underweight** | 0.156 | -0.175 | 0.069 | 0.272 | 0.881 |
| **Number chronic health conditions** | 0.047 | 0.168 | -0.596 | -0.003 | 0.623 |
| **Symptoms of illness past 30 days** | -0.064 | 0.183 | -0.442 | 0.173 | 0.746 |
| **HIV** | -0.207 | 0.156 | -0.152 | 0.237 | 0.875 |
| **Walls cement/bricks** | 0.671 | -0.077 | 0.007 | -0.044 | 0.583 |
| **Roof cement/bricks/tiles** | 0.309 | 0.407 | -0.110 | -0.129 | 0.612 |
| **Water ladder** | 0.721 | 0.040 | 0.021 | 0.080 | 0.443 |
| **Sanitation ladder** | 0.515 | 0.054 | -0.039 | 0.017 | 0.714 |
| **Frequency of crimes in neighbourhood** | -0.041 | 0.153 | -0.012 | 0.049 | 0.978 |
| Quartimin rotated factor loadings. Factor loadings >0.32 are highlighted in grey. The uniqueness differs here from the unrotated factor loadings as we retained 4 factors and not 5. | | | | | |

Table A5 Factor scoring coefficients computed using regression method

|  | (1) | (2) | (3) | (4) |
| --- | --- | --- | --- | --- |
|  | **Living conditions** | **Physical Health** | **Soc. econ. status** | **Life styles** |
|  |  |  |  |  |
| **Social ladder** | 0.011 | 0.151 | 0.041 | -0.119 |
| **Death of HH-member past 2 years** | -0.025 | 0.028 | 0.013 | -0.013 |
| **Importance of religious activities** | 0.016 | 0.068 | -0.049 | -0.030 |
| **Area preferences** | -0.046 | 0.015 | 0.095 | 0.030 |
| **Education** | 0.039 | 0.131 | 0.360 | 0.012 |
| **Material index** | 0.137 | 0.215 | 0.039 | -0.067 |
| **Monthly per capita HH-consumption (log)** | 0.037 | 0.27 | 0.022 | 0.048 |
| **Physical activity** | 0.008 | 0.081 | 0.167 | 0.143 |
| **Number daily cigarettes** | 0.032 | -0.007 | -0.002 | 0.281 |
| **Number daily units of alcohol** | 0.028 | -0.037 | 0.016 | 0.224 |
| **BMI underweight** | 0.038 | -0.071 | 0.020 | 0.176 |
| **Number chronic health conditions** | 0.003 | 0.112 | -0.311 | 0.000 |
| **Symptoms of illness past 30 days** | -0.024 | 0.103 | -0.201 | 0.137 |
| **HIV** | -0.055 | 0.062 | -0.071 | 0.174 |
| **Walls cement/bricks** | 0.28 | -0.036 | 0.028 | -0.048 |
| **Roof cement/bricks/tiles** | 0.13 | 0.273 | -0.071 | -0.113 |
| **Water ladder** | 0.381 | 0.066 | 0.034 | 0.097 |
| **Sanitation ladder** | 0.177 | 0.043 | -0.016 | 0.007 |
| **Frequency of crimes in neighbourhood** | -0.011 | 0.061 | -0.008 | 0.033 |
| Predictions of factors made using regression method based on quartimin rotated factors. Variable means 0 and standard deviations 1 assumed. | | | | |

**Table A6 1st stage estimates of the reduced sample baseline mediator control estimations**

|  | (1) | (2) | (3) | (4) | (5) |
| --- | --- | --- | --- | --- | --- |
|  | Mediator equations: No Baseline Mediators | Life styles equation:  Baseline  Life styles | Physical health equation:  Baseline Physical health | CES-D equation:  No Baseline Mediators | CES-D equation:  Baseline Mediators |
|  |  |  |  |  |  |
| HH with eligible child | 0.738*** | 0.738*** | 0.738*** | 0.740*** | 0.740*** |
|  | (0.016) | (0.016) | (0.016) | (0.016) | (0.016) |
| Life styles at baseline |  | 0.001 |  |  | -0.000 |
|  |  | (0.003) |  |  | (0.003) |
| Physical health at |  |  | -0.007 |  | -0.000 |
| baseline |  |  | (0.013) |  | (0.015) |
| Constant | 0.165** | 0.166** | 0.178** | 0.267*** | 0.267*** |
|  | (0.065) | (0.065) | (0.071) | (0.095) | (0.096) |
|  |  |  |  |  |  |
| Province and region at | YES | YES | YES | YES | YES |
| baseline  Year | YES | YES | YES | YES | YES |
| Observations | 3,420 | 3,420 | 3,420 | 3,420 | 3,420 |
| R-squared | 0.515 | 0.515 | 0.515 | 0.516 | 0.516 |
| Clustered standard errors in parenthesis; *** p<0.01, ** p<0.05, * p<0.1. We present here the first stage estimation results of the instrumental variable estimation. The instrumented variable is HH receives CSG which is instrumented by HH with an eligible child, which is the outcome variable in model (1) - (5). We control baseline covariates in the estimations. | | | | | |

**Table A7 2^nd^ stage estimates of reduce sample baseline mediator control estimations**

|  | (1) | (2) | (3) | (4) | (5) | (6) |
| --- | --- | --- | --- | --- | --- | --- |
|  | Life styles equation:  No Baseline Life styles | Life styles equation: Baseline Life styles | Physical health equation:  No Baseline Physical health | Physical health equation: Baseline Physical health | CES-D equation:  No Baselines Mediators | CES-D equation: Baseline Mediators |
|  |  |  |  |  |  |  |
| HH receives CSG | -0.346*** | -0.260** | 0.071* | 0.047 | 0.732*** | 0.727*** |
| Life styles  Physical health | (0.126) | (0.114) | (0.037) | (0.033) | (0.245)  -0.161***  (0.046)  0.993***  (0.146) | (0.244)  -0.159***  (0.045)  0.965***  (0.149) |
| Life styles at baseline |  | 0.228*** |  |  |  | -0.011 |
|  |  | (0.042) |  |  |  | (0.035) |
| Physical health at |  |  |  | 0.353*** |  | 0.071 |
| baseline |  |  |  | (0.023) |  | (0.150) |
| Constant | 0.737** | 0.910*** | 1.644*** | 1.042*** | 19.514*** | 19.467*** |
|  | (0.350) | (0.332) | (0.102) | (0.102) | (0.966) | (0.980) |
|  |  |  |  |  |  |  |
| Province and region at | YES | YES | YES | YES | YES | YES |
| baseline  Year | YES | YES | YES | YES | YES | YES |
| Observations | 3,420 | 3,420 | 3,420 | 3,420 | 3,420 | 3,420 |
| R-squared | 0.153 | 0.203 | 0.437 | 0.511 | 0.100 | 0.100 |
| Clustered standard errors in parenthesis; *** p<0.01, ** p<0.05, * p<0.1. We present here the second stage estimation results of the instrumental variable estimation. The instrumented variable is HH receives CSG which is instrumented by HH with an eligible child. We control for baseline covariates in the estimation. The outcome variable in (1) and (2) is life styles, in (3) and (4) it is physical health and in (5) and (6) it is CES-D. | | | | | | |

**Table A8 Error correlations of mediator and outcome equations**

|  | **Residual Outcome equation** | **Residual Physical health equation** | **Residual Life styles equation** |
| --- | --- | --- | --- |
| **(1) Residual Outcome equation** | 1 |  |  |
| **(2) Residual Physical health equation** | 0.0005 | 1 |  |
| **(3) Residual Life styles equation** | -0.0009 | 0.0155 | 1 |
| Correlation tests of residuals from (1) the outcome equation, (2) the mediator equation with Physical health as dependent variable, (3) the mediator equation with Life styles as dependent variable. | | | |

**Table A9 Tests of mediator-mediator and mediator-treatment interaction effects on CES-D**

|  | (1) | (2) | (3) | (4) |
| --- | --- | --- | --- | --- |
|  | CES-D | CES-D | CES-D | CES-D |
|  |  |  |  |  |
| Living conditions | 0.648*** | 0.643*** | 0.640*** | 0.644*** |
|  | (0.185) | (0.184) | (0.184) | (0.184) |
| Physical health | 1.023*** | 1.016*** | 1.112*** | 1.128*** |
|  | (0.151) | (0.159) | (0.182) | (0.182) |
| Physical health*Life styles | -0.002 |  |  |  |
|  | (0.034) |  |  |  |
| Soc. econ. Status | -0.102 | -0.098 | -0.101 | -0.103 |
|  | (0.136) | (0.134) | (0.135) | (0.135) |
| Life styles | -0.180*** | -0.171*** | -0.144*** | -0.141*** |
|  | (0.058) | (0.049) | (0.052) | (0.051) |
| HH receives CSG | 0.710*** | 0.654*** |  |  |
|  | (0.213) | (0.184) |  |  |
| Interact: Physical health*HH receives CSG |  | 0.011 |  |  |
|  |  | (0.166) |  |  |
| Interact: Life styles*HH receives CSG |  | -0.026 |  |  |
|  |  | (0.060) |  |  |
| HH with eligible child |  |  | 0.732*** |  |
|  |  |  | (0.197) |  |
| Interact: Physical health*HH with eligible child |  |  | -0.164 |  |
|  |  |  | (0.196) |  |
| Interact: Life styles*HH with eligible child |  |  | -0.070 |  |
|  |  |  | (0.059) |  |
| IV( HH receives CSG) |  |  |  | 1.007*** |
|  |  |  |  | (0.261) |
| Interact: Physical health*IV(HH receives CSG) |  |  |  | -0.244 |
|  |  |  |  | (0.247) |
| Interact: Life styles*IV(HH receives CSG) |  |  |  | -0.101 |
|  |  |  |  | (0.075) |
| Constant | 19.654*** | 19.685*** | 19.675*** | 19.600*** |
|  | (0.863) | (0.858) | (0.859) | (0.860) |
|  |  |  |  |  |
| Province and region at baseline | YES | YES | YES | YES |
| Year | YES | YES | YES | YES |
| Wald test joint interaction (Prob. > F) |  | 0.912 | 0.315 | 0.219 |
| Observations | 4,535 | 4,535 | 4,535 | 4,535 |
| R-squared | 0.095 | 0.095 | 0.093 | 0.093 |
| Clustered standard errors in parentheses; *** p<0.01, ** p<0.05, * p<0. The outcome variable is CES-D. The variable IV (HH receives CSG) in model (4) is the instrumented HH CSG with HH with eligible child. Model (4) presents the second stage estimation results of the 2SLS estimation. I present here only the main variables of interest but control at any time for baseline covariates. | | | | |

**Table A10 Placebo test estimations 2SLS with mediators at baseline and transfer and eligibility from the first round**

|  | (1) | (2) | (3) | (4) |
| --- | --- | --- | --- | --- |
|  | 1st Stage:  Life styles at baseline | 2nd Stage:  Life styles at baseline | 1st Stage: Physical health at baseline | 1st Stage: Physical health at baseline |
|  |  |  |  |  |
| HH receives CSG in t+1 |  | -0.185 |  | 0.069 |
|  |  | (0.158) |  | (0.048) |
| HH with eligible child in t+1 | 0.688*** |  | 0.688*** |  |
|  | (0.023) |  | (0.022) |  |
| Soc. econ. status at baseline | -0.023 | -1.821*** | -0.007 | -0.245*** |
|  | (0.019) | (0.180) | (0.023) | (0.036) |
| Physical health at baseline | -0.021 | -0.601*** |  |  |
|  | (0.017) | (0.093) |  |  |
| Living conditions at baseline | -0.031 | 2.545*** | -0.055* | 0.441*** |
|  | (0.025) | (0.234) | (0.031) | (0.044) |
| Life styles at baseline |  |  | 0.008 | -0.064*** |
|  |  |  | (0.006) | (0.011) |
| Constant | 0.324** | 1.405** | 0.286** | 1.649*** |
|  | (0.126) | (0.546) | (0.121) | (0.162) |
|  |  |  |  |  |
| Province and region at baseline | YES | YES | YES | YES |
| Year | YES | YES | YES | YES |
| Observations | 1,830 | 1,830 | 1,830 | 1,830 |
| R-squared | 0.448 | 0.560 | 0.448 | 0.499 |
| Clustered standard errors in parentheses; *** p<0.01, ** p<0.05, * p<0. We use here only the second wave, year 2010 for the variables HH receives CSG and HH with eligible child with controls and mediators and outcome at baseline The outcome variable in the first stage is the binary variable HH CSG. The outcome variable in the second stage is CES-D. We control for baseline covariates in the estimations. Model (1) is the first stage estimation of the life styles estimation with HH receives CSG as the outcome variable, (2) is the second stage estimation with life styles as the outcome variable. Model (3) is the first stage estimation of the physical health estimation with HH receives CSG as the outcome variable, (4) is the second stage estimation with physical health as the outcome variable. | | | | |

**Table A11 Estimation of the direct effect equation and mediation equations controlling for child age effects on mental health**

|  | (1) | (2) | (3) | (4) | (5) | (6) | (7) |
| --- | --- | --- | --- | --- | --- | --- | --- |
|  | First Stage: HH receives CSG | 2nd Stage: Direct effect | 2nd Stage: Living conditions | 2nd Stage: Soc. econ. status | 2nd Stage: Phys.  Health | 2nd Stage: Life styles | 2nd Stage: Mediation |
|  |  |  |  |  |  |  |  |
| HH receives CSG |  | 0.874*** | -0.001 | 0.110** | 0.057* | -0.402*** | 0.752*** |
|  |  | (0.236) | (0.036) | (0.044) | (0.033) | (0.114) | (0.228) |
| HH with CSG eligible child | 0.731*** |  |  |  |  |  |  |
|  | (0.016) |  |  |  |  |  |  |
| Living conditions |  |  |  |  |  |  | 0.653*** |
|  |  |  |  |  |  |  | (0.183) |
| Socio economic status |  |  |  |  |  |  | -0.077 |
|  |  |  |  |  |  |  | (0.132) |
| Physical |  |  |  |  |  |  | 1.020*** |
|  |  |  |  |  |  |  | (0.138) |
| Life styles |  |  |  |  |  |  | -0.181*** |
|  |  |  |  |  |  |  | (0.042) |
|  |  |  |  |  |  |  |  |
| Constant | 0.092* | 22.500*** | 2.428*** | 3.564*** | 1.723*** | 0.570** | 19.535*** |
|  | (0.055) | (0.625) | (0.113) | (0.112) | (0.089) | (0.280) | (0.858) |
| Year | YES | YES | YES | YES | YES | YES | YES |
| Province and region at baseline | YES | YES | YES | YES | YES | YES | YES |
| Covariates at baseline | YES | YES | YES | YES | YES | YES | YES |
| Age of cohabiting children | YES | YES | YES | YES | YES | YES | YES |
| Observations | 4,535 | 4,535 | 4,535 | 4,535 | 4,535 | 4,535 | 4,535 |
| R-squared | 0.531 | 0.070 | 0.364 | 0.135 | 0.440 | 0.156 | 0.100 |
| Clustered standard errors in parentheses; *** p<0.01, ** p<0.05, * p<0. The instrumented variable is HH receives CSG which is instrumented by HH with an eligible child. (1) is the first stage estimation of the 2SLS. The outcome variable is HH receives CSG. (2) to (7) are second stage estimations of the 2SLS. The outcome variable in (2) is CES-D, in (3) it is Living conditions, in (4) it is Socio economic status, in (5) Physical health, (6) Life styles, and in (7) it is CES-D. | | | | | | | |

**Table A12 Logistic regression estimation of attrition at various stages on baseline characteristics**

|  | (1) | (2) | (3) |
| --- | --- | --- | --- |
|  | All waves and baseline vs rest | 2 waves and baseline vs rest | 1 wave and baseline vs rest |
|  |  |  |  |
| Economic decision maker | 0.130 | -0.004 | -0.028 |
|  | (0.160) | (0.084) | (0.081) |
| Male | -0.476*** | -0.246*** | 0.343*** |
|  | (0.137) | (0.069) | (0.066) |
| Age | 0.014 | 0.008 | -0.009 |
|  | (0.023) | (0.012) | (0.011) |
| Age Squared | -0.000 | -0.000 | 0.000 |
|  | (0.000) | (0.000) | (0.000) |
| Under 19 years of age | -0.124 | 0.058 | -0.015 |
|  | (0.216) | (0.118) | (0.113) |
| HH receives Old Age Pension | 0.110 | 0.057 | -0.081 |
|  | (0.151) | (0.082) | (0.078) |
| HH receives Disability Grant | -0.257 | 0.054 | 0.011 |
|  | (0.212) | (0.102) | (0.098) |
| HH receives Foster Care Grant | 0.388 | 0.170 | -0.268* |
|  | (0.280) | (0.166) | (0.159) |
| HH receives Care Dependency Grant | -0.466 | 0.385 | -0.267 |
|  | (0.734) | (0.301) | (0.297) |
| Number of HH members | 0.009 | 0.017 | -0.018 |
|  | (0.029) | (0.016) | (0.015) |
| Tribal authority area | 0.035 | 0.110 | -0.110 |
|  | (0.228) | (0.125) | (0.119) |
| Urban formal | -0.257 | 0.037 | 0.030 |
|  | (0.237) | (0.125) | (0.119) |
| Urban informal | -0.452 | 0.017 | 0.096 |
|  | (0.325) | (0.166) | (0.158) |
| Limpopo | -0.600* | -0.154 | 0.284* |
|  | (0.324) | (0.163) | (0.155) |
| Western Cape | -0.058 | 0.073 | -0.046 |
|  | (0.219) | (0.128) | (0.122) |
| Eastern Cape | -0.465 | -0.290* | 0.382** |
|  | (0.332) | (0.173) | (0.165) |
| Northern Cape | -0.459 | -0.036 | 0.150 |
|  | (0.341) | (0.173) | (0.166) |
| Free State | -0.095 | 0.078 | -0.043 |
|  | (0.196) | (0.115) | (0.110) |
| Kwazulu-West | -0.923** | 0.173 | 0.043 |
|  | (0.400) | (0.171) | (0.166) |
| North West | 0.040 | -0.003 | -0.000 |
|  | (0.295) | (0.168) | (0.159) |
| Gauteng | -0.521 | 0.085 | 0.057 |
|  | (0.333) | (0.167) | (0.161) |
|  |  |  |  |
| Constant | -2.288*** | -1.016*** | 0.576** |
|  | (0.536) | (0.285) | (0.273) |
| Observations | 4,535 | 4,535 | 4,535 |
| Robust clustered standard errors in parentheses; *** p<0.01, ** p<0.05, * p<0.1. We estimate three logistic regression models. The outcome in the first model is a binary variable taking value one for individuals that are observed at any point and zero otherwise. The outcome in the second model is a binary variable taking value one for individuals that are observed at baseline and twice thereafter and zero otherwise. The outcome in the third model is a binary variable taking value one for individuals that are observed at baseline and once thereafter and zero otherwise. | | | |

**Table A13 Logistic regression estimation of attrition at various stages on 19 baseline mediator items and baseline characteristics**

|  | (1) | (2) | (3) |
| --- | --- | --- | --- |
|  | All waves and baseline vs rest | 2 waves and baseline vs rest | 1 wave and baseline vs rest |
|  |  |  |  |
| BMI underweight | 0.200 | 0.062 | -0.111 |
|  | (0.228) | (0.124) | (0.119) |
| Nb. cigarettes | 0.003 | -0.004 | 0.002 |
|  | (0.022) | (0.011) | (0.011) |
| Nb. units of alcohol | 0.005 | -0.001 | -0.001 |
|  | (0.011) | (0.006) | (0.006) |
| Consumption | -0.196* | -0.003 | 0.053 |
|  | (0.115) | (0.060) | (0.057) |
| HIV | -1.362 | 0.185 | 0.046 |
|  | (1.030) | (0.309) | (0.306) |
| Death HH member | -0.366* | -0.004 | 0.099 |
|  | (0.208) | (0.101) | (0.098) |
| Walls cement/brick | -0.046 | -0.130 | 0.135 |
|  | (0.175) | (0.095) | (0.091) |
| Roof cement/bricks/tiles | 0.126 | -0.172 | 0.126 |
|  | (0.374) | (0.206) | (0.194) |
| Social ladder | 0.057 | -0.026 | 0.012 |
|  | (0.089) | (0.047) | (0.045) |
| Area preferences | -0.035 | -0.057* | 0.060* |
|  | (0.062) | (0.033) | (0.031) |
| Religious activities | -0.049 | -0.009 | 0.022 |
|  | (0.098) | (0.053) | (0.050) |
| Education | 0.065 | 0.012 | -0.030 |
|  | (0.132) | (0.069) | (0.066) |
| Material index | 0.017 | -0.047 | 0.038 |
|  | (0.070) | (0.037) | (0.036) |
| Physical activity | -0.006 | -0.059 | 0.056 |
|  | (0.081) | (0.043) | (0.041) |
| Number chronic conditions | -0.702*** | -0.079 | 0.192** |
|  | (0.215) | (0.077) | (0.076) |
| Number symptoms of illness | -0.007 | -0.007 | 0.008 |
|  | (0.046) | (0.023) | (0.022) |
| Water ladder | 0.120 | -0.039 | 0.003 |
|  | (0.133) | (0.073) | (0.070) |
| Sanitation ladder | 0.123 | 0.053 | -0.081** |
|  | (0.079) | (0.043) | (0.041) |
| Constant | -1.775* | -0.659 | 0.115 |
|  | (1.059) | (0.560) | (0.538) |
|  |  |  |  |
| Province and region at baseline | YES | YES | YES |
| Observations | 3,420 | 3,420 | 3,420 |
| Robust clustered standard errors in parentheses; *** p<0.01, ** p<0.05, * p<0.1. We estimate three logistic regression models on a smaller sample for missing information in baseline mediator variables. The outcome in the first model is a binary variable taking value one for individuals that are observed at any point and zero otherwise. The outcome in the second model is a binary variable taking value one for individuals that are observed at baseline and twice thereafter and zero otherwise. The outcome in the third model is a binary variable taking value one for individuals that are observed at baseline and once thereafter and zero otherwise. Highlighted in grey are those mediator baseline variables which show a significant association with the outcome. | | | |


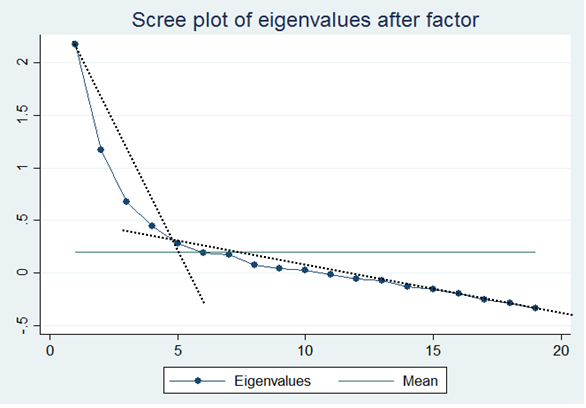


**Figure A1 Scree plot of eigenvalues of the unrotated Exploratory Factor Analysis**

**APPENDIX: References**

Bartlett, M. . (1937) ‘Properties of Sufficiency and Statistical Tests.’ *Proceedings of the Royal Society of London, Series A. Mathematical and Physical Sciences*, 160(901) pp. 268–282.

Cerny, B. A. and Kaiser, H. F. (1977) ‘A Study Of A Measure Of Sampling Adequacy For Factor-Analytic Correlation Matrices.’ *Multivariate Behavioral Research*. Routledge, 12(1) pp. 43–47.

Eyal, K. and Burns, J. (2015) *Up or Down? Intergenerational Mental Health Transmission and Cash Transfers in South Africa*. (Working Paper).

Kaiser, H. F. (1974) ‘An index of factorial simplicity.’ *Psychometrika*, 39(1) pp. 31–36.

Tabachnick, B. G. and Fidell, L. S. (2007) *Using Multivariate Statistics*. 5th ed., Pearson Allyn & Bacon.

Yong, A. G. and Pearce, S. (2013) ‘A Beginner ’ s Guide to Factor Analysis : Focusing on Exploratory Factor Analysis.’ *Tutorials in Quantitative Methods for Psychology*, 9(2) pp. 79–94.
